# Predictors of Daily Mobility of Adults in Peri-Urban South India

**DOI:** 10.3390/ijerph14070783

**Published:** 2017-07-14

**Authors:** Margaux Sanchez, Albert Ambros, Maëlle Salmon, Santhi Bhogadi, Robin T. Wilson, Sanjay Kinra, Julian D. Marshall, Cathryn Tonne

**Affiliations:** 1 Centre for Research in Environmental Epidemiology (CREAL), ISGlobal, 08003 Barcelona, Spain; albert.ambros@isglobal.org (A.A.); maelle.salmon@isglobal.org (M.S.); cathryn.tonne@isglobal.org (C.T.); 2 Universitat Pompeu Fabra, 08002 Barcelona, Spain; 3 CIBER Epidemiología y Salud Pública (CIBERESP), 28029 Madrid, Spain; 4 Public Health Foundation of India, New Delhi 110070 e, India; kammilisanthi@gmail.com; 5 Geography & Environment, University of Southampton, Highfield Campus, Southampton SO17 1BJ, UK; robin@rtwilson.com; 6 Department of Non-communicable Disease Epidemiology, London School of Hygiene and Tropical Medicine, London WC1E 7HT, UK; sanjay.kinra@lshtm.ac.uk; 7 Department of Civil and Environmental Engineering, University of Washington, Seattle, WA 98195, USA; marshall.julian@gmail.com

**Keywords:** gender, global positioning system (GPS), India, principal component analysis (PCA), spatial behavior, time-activity patterns

## Abstract

Daily mobility, an important aspect of environmental exposures and health behavior, has mainly been investigated in high-income countries. We aimed to identify the main dimensions of mobility and investigate their individual, contextual, and external predictors among men and women living in a peri-urban area of South India. We used 192 global positioning system (GPS)-recorded mobility tracks from 47 participants (24 women, 23 men) from the Cardiovascular Health effects of Air pollution in Telangana, India (CHAI) project (mean: 4.1 days/person). The mean age was 44 (standard deviation: 14) years. Half of the population was illiterate and 55% was in unskilled manual employment, mostly agriculture-related. Sex was the largest determinant of mobility. During daytime, time spent at home averaged 13.4 (3.7) h for women and 9.4 (4.2) h for men. Women’s activity spaces were smaller and more circular than men’s. A principal component analysis identified three main mobility dimensions related to the size of the activity space, the mobility in/around the residence, and mobility inside the village, explaining 86% (women) and 61% (men) of the total variability in mobility. Age, socioeconomic status, and urbanicity were associated with all three dimensions. Our results have multiple potential applications for improved assessment of environmental exposures and their effects on health.

## 1. Introduction

How and where people spend their time can strongly influence environmental exposures relevant to health. Time-activity patterns have been identified as one of the main sources of exposure measurement error in air pollution epidemiology [1]. Recent studies highlighted the importance of integrating mobility for better air pollution exposure assessment [2,3,4,5,6]. However, existing literature describing time-activity or mobility patterns and their determinants is largely from populations in urban areas of high-income countries [7,8,9]. Little evidence is available regarding individual or contextual determinants of mobility for populations experiencing rapid economic development and urbanization. Findings from high-income countries may not be relevant to populations in low- and middle-income countries. The gap in knowledge is especially relevant for populations in peri-urban areas of India experiencing urbanization, which may not be well represented by the most recent national time-use survey from 1998 to 1999 [10].

Global positioning system (GPS) technologies provide an alternative to self-reported data gathered via surveys or diaries to investigate daily mobility or time-activity patterns [11]. The shortcomings of GPS (large amount of data, presence of missing data, battery and coverage issues) are outweighed by advantages such as low-cost, objectivity, low burden for participants, and high temporal precision. Automatic algorithms now detect locations, trips, and transportation modes from GPS tracks [12,13,14,15]. GPS data have been used to investigate the impact of human mobility in infectious disease dynamics [16], exposure to food environment [17], levels of physical activity and sedentary behavior [18,19], and to reduce misclassification in outdoor air pollution exposure assessment as compared to home-based estimates [2,3].

The multidimensional nature of mobility makes it difficult to characterize thoroughly, while at the same time parsimoniously. Some previous studies have identified the main dimensions of mobility across multiple indicators based on a priori hypotheses or data-driven reduction techniques [20,21,22], thereby facilitating the analysis of daily mobility in health studies. As part of the CHAI (Cardiovascular Health effects of Air pollution in Telangana, India) project, we collected daytime GPS tracks and time-activity diaries for one to six days per individual, during May 2015 to February 2016, for 60 adults living in a peri-urban area near Hyderabad, India. Our overall objective was to characterize objectively measured daily mobility of adults and explore how mobility differs between men and women. Specifically, we identified 1/the main dimensions of mobility across several indicators using principal component analysis, a data reduction technique, and 2/individual, external, and contextual predictors of these mobility dimensions.

## 2. Materials and Methods

### 2.1. Study Design

We used data collected through the CHAI project (www.chaiproject.org), nested in the Andhra Pradesh Children and Parents Study (APCAPS) cohort [23]. Briefly, APCAPS is a large prospective, intergenerational cohort study including ~6000 participants living near the city of Hyderabad, India. The study area consists of 28 villages each with 187 to 5065 households spread over 543 km^2^ southeast of Hyderabad (mean distance from the city center of Hyderabad: 28 km). Initiated in 2015, CHAI included detailed measurements of time-activity and particulate air pollution exposure for a sex- and village-matched subset of APCAPS participants (*n* = 402) [24]. A sex- and village-matched subsample of 60 willing participants was further invited to participate in a panel study consisting of several GPS collection sessions over the course of one year. Ethics approval for the CHAI project was granted by the Ethics Committees of Parc de Salut MAR (Barcelona, Spain), the Indian Institute of Public Health-Hyderabad (Hyderabad, India), and the National Institute of Nutrition (Hyderabad, India).

### 2.2. GPS Data Collection and Processing

Participants wore a secured backpack containing a GPS receiver (Etrex 20; Garmin, Inc., Lenexa, KS, USA) for one 24-h session every two months, up to 6 sessions, between May 2015 and February 2016. GPS receivers had 4-m accuracy in the study area. Sessions began with a field worker setting up the receiver at the participant’s house (average time: 8 a.m.) and finished the following day around the same time with a field worker. The beginning and ending time were chosen such as the participant did not need to adapt his/her daily activities. Sessions were planned according to participant availability and included week days, as well as weekend days. There was no difference in the monitoring procedure according to day type. Date, time, latitude, and longitude were recorded at a 30-s interval by the GPS device during the whole session day. Participants were asked to wear the backpack during their daytime activities. If the backpack interfered with activities (e.g., sleeping, sitting), participants were instructed to place the backpack nearby.

Here, we investigated daytime (6 a.m. to 10 p.m.) data only, given that most of the nighttime was spent within 100-m of the home in our population. Abrupt position changes resulting in >1 km separation between two points (namely, speed >120 km/h) were identified as outliers and removed. Cold start position acquisition (points >50 m from the house at the beginning of the session) were removed. We considered a track as invalid if the participant did not carry the device (i.e., the collocated accelerometer recorded no motion during the session day—poor compliance) or if the GPS device failed to record at least 13.9 h (being 87% of the expected daytime duration).

### 2.3. Study Population

A total of 246 GPS tracks were collected for the selected 60 participants. Thirteen participants showed poor compliance at the first session and dropped out. In the present analysis, we analyzed data from 47 unique participants with 192 valid GPS monitoring days (on average, 4.1 days per participant). Excluded participants were slightly younger and more likely to be unemployed (housewife, students, retired, or people not seeking work) than included participants (Supplementary Table S1).

### 2.4. Time-Activity Diary

After each session, participants completed an hourly time-activity diary under supervision of field staff. Participants reported hourly main location (e.g., indoor at home, outdoor in village) and main activities (e.g., cooking, sleeping, working) for the prior 24 h.

### 2.5. Geographic Information System (GIS)-Derived Data

As part of the APCAPS and CHAI studies, the following locations in the study area were geocoded: participants’ residence (entrance door); nearby non-residential places (e.g., commercial services, shops, temples); industrial places with regular operation, and; the main entrance of the Rajiv Gandhi International Airport. Roads throughout the study area were mapped by tracing satellite imagery in OpenStreetMap and confirmed through visits by the field team. The centroid of the residence village was derived using the village built surface area. The night-time light intensity in 2012 over the built area was used as a village-level marker of economic activity and urbanicity. The night-time light intensity ranges from 0 (no light) to 63 (high intensity) and is derived from measurements of light emissions from persistent sources associated with human settlement provided by the Defense Meteorological Satellite Program satellites [25,26].

### 2.6. Other Data

Individual—Socio-demographic data collected in the CHAI baseline questionnaire prior to GPS measurements included marital status, primary occupation, education level, tobacco use, and household motorcycle and bicycle ownership. Age was calculated at 1 May 2015. Body mass index in kg/m^2^ was collected in the third APCAPS follow-up during 2010–2012.

External—We defined season as follows: summer (March to May 2015; typically high temperature), monsoon (June to August 2015; typically high humidity), post-monsoon (September to November 2015), and winter (December 2015 to February 2016). Weekends are Saturdays and Sundays.

Village-level—Based on data collected in the third APCAPS follow-up, we derived village-level indicators of solid fuel use, ownership of motorcycles, and ownership of bicycles.

### 2.7. Mobility Indicators

Existing literature employs multiple metrics for mobility; we therefore considered several indicators for each GPS track (Table 1). We calculated the mean and median linear distance from home, as well as daytime spent within village boundaries and within 50, 100, 400, 800, and 1600 m of home. Two classical activity spaces were considered: the minimum convex hull and the 1-standard deviational ellipse; we derived their surface, perimeter, centroid-to-home distance, and compactness (i.e., how circular the activity space is). We identified the residential place and activity locations by applying an automated algorithm for detecting clusters of points employing selected space (10 m) and time (30 min) thresholds [15]. Points between locations, namely not part of an identified cluster, either residential or not, were considered as trips. We derived (1) the time spent at residential place, in activity locations, and in trips; (2) the total number of activity locations, the number of activity locations within the 1-standard deviational ellipse, and their distance from home; and (3) the number of trips ≥5 min and the average speed during trips (Table 1).

### 2.8. Analyses

Sex was the only individual factor consistently and significantly associated with mobility in our population (Supplementary Figure S1). Sex was also highly correlated with several other individual characteristics (e.g., most illiterate participants were women). Given the strong effect of sex on mobility, its high correlation with other characteristics, along with its relevance as a determinant of health, health behaviors, and environmental exposures, we performed analyses stratified by sex.

The balance of within- and between-participant variance in each indicator was quantified using the intra-class correlation coefficient (ICC). As the ICC approaches 0, within-participant variability (i.e., over time) dominates; as ICC approaches 1, between-participant variability dominates. We identified the main independent dimensions of daily mobility among the 25 available mobility indicators (Table 1) using a principal component analysis with Varimax rotation on the 192 GPS tracks. The principal component analysis aims to reduce the number of observed indicators into a smaller number of independent dimensions that account for the largest possible variability among the indicators. The number of retained dimensions is based on the percentage of total variability they explained as well as their interpretability. A score was attributed to each participant/day for each of the identified dimensions. To investigate the sensitivity of the mobility dimensions to the repeated design, we performed a sensitivity analysis considering only the first GPS session, decreasing the number of tracks used from 192 to 47.

We investigated differences between groups using χ^2^ test (categorical variables) or analysis of variance (ANOVA, continuous variables). Predictors of mobility indicators or mobility dimension scores derived from principal component analysis were investigated through mixed models with random intercept per participant, in order to take into account the repeated nature of the data. GPS- and GIS-derived data were processed using ArcGIS platform (v10.2.1, ESRI, Redlands, CA, USA) with SpatiaLite (v4.1.1) and QGIS (v2.12.3, QGIS Development Team, Lyon, France). Statistical analyses were conducted in R version 3.2.2 (R Foundation for Statistical Computing, Vienna, Austria) [27] using several packages [28,29,30,31,32].

## 3. Results

The characteristics of the study population are presented in Table 2. All participants reported Hindu religion. The mean (standard deviation, sd) age of the study population was 44 (14) years, with women being older than men. Women reported mostly unskilled manual occupation (71% vs. 39% for men), largely related to agriculture, and were less educated then men (79% and 26% illiteracy, respectively). Mean (sd) distance between residence and the nearest primary road was 4.4 (2.8) km. Other household characteristics along with village characteristics are presented in Supplementary Table S2.

**Figure 1:**
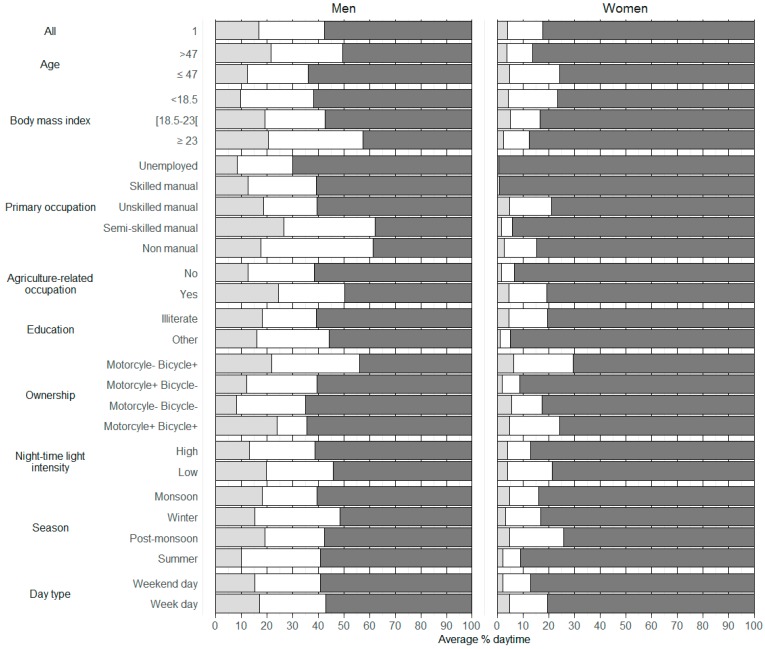
Percent of daytime spent at home (dark grey), in activity locations (white) and trips (light grey) according to selected characteristics. Home, activity locations, and trips identified by an automated algorithm within GPS tracks. Body mass index is expressed in kg/m^2^. Night-time light intensity was used as marker of village urbanicity. Low and high categories for night-time light intensity were derived from population median value. All tests comparing men and women values were significant at the 5% level.

During daytime, participants spent an average (sd) of 11.5 (4.4) h at home, 3.2 (3.5) h in activity locations, and 1.6 (2.0) h in trips, but with major differences by sex. Women spent an average (sd) of 13.4 (3.7) h at home, compared to 9.4 (4.2) h for men (*p* < 0.01), representing 83% and 57%, respectively, of their daytime (Figure 1). Women spent six times more time at home than in other activity locations, while the corresponding ratio for men was only two. Mean daytime spent in trips was 2.8 h for men vs. only 0.6 h for women. Men made more trips than women (mean (sd) number of daily trips ≥5 min: 4.6 (3.5) vs. 1.5 (1.9), respectively) and men visited more activity locations than women (Table 3). Women’s activity spaces were much smaller, more circular, and more home-centered than men’s (Supplementary Table S3). Variability over time (i.e., within-participant) predominated for almost all mobility indicators. The most variable mobility indicators related to surface, perimeter, and centroid-to-home distance of the activity space with ICC ranging from 0.12 to 0.30, mainly because of male participants (Supplementary Table S4).

In the study population, 72% and 14% of the points identified as “time spent at home” by the automated algorithm were consistently self-reported as “indoor at home” and “in playground or compound” in the hourly time-activity diary. This overall level of agreement was higher in women (76% and 16%, respectively) than in men (66% and 11%, respectively). In the hourly time-activity diary, men reported more “work” during daytime than women (27% vs. 15%, respectively). However, a similar proportion of points (~58%) identified as out of the residential place (namely, in activity places or in trips) by the automated algorithm were reported as “work” or “travel” by men and women.

The principal component analysis identified five and three dimensions of mobility, explaining 80% and 86% of the total variability in mobility for men and women, respectively (Table 4). We labeled the dimensions according to the meaning of the indicators with the largest contribution. The size of the activity space dimension, with high contribution of the surface and perimeter of the activity space, was relevant for both men and women, but explained more variation in women (42%) than men (24%). Among men, the mobility in and around home dimension together with a dimension related to the circularity of the activity space explained 37% of the total variability in mobility. Among women, these two dimensions were grouped and explained a similar proportion of the total variability. Another common dimension to both sexes was that of mobility inside village, with a strong positive contribution of the proportion of activity locations visited inside village boundaries (Table 4). These locations were most likely reached by walking by women (negative contribution of trip speed). The last dimension of men’s mobility explained almost 10% of the total variability, with the highest contribution of the median distance travelled from home. The results of the principal component analysis in men and women combined were similar to those found in men only and explained 84% of the total variability in mobility (Supplementary Table S5). Using only data from the first session led to comparable mobility dimensions (Supplementary Table S6).

**Figure 2:**
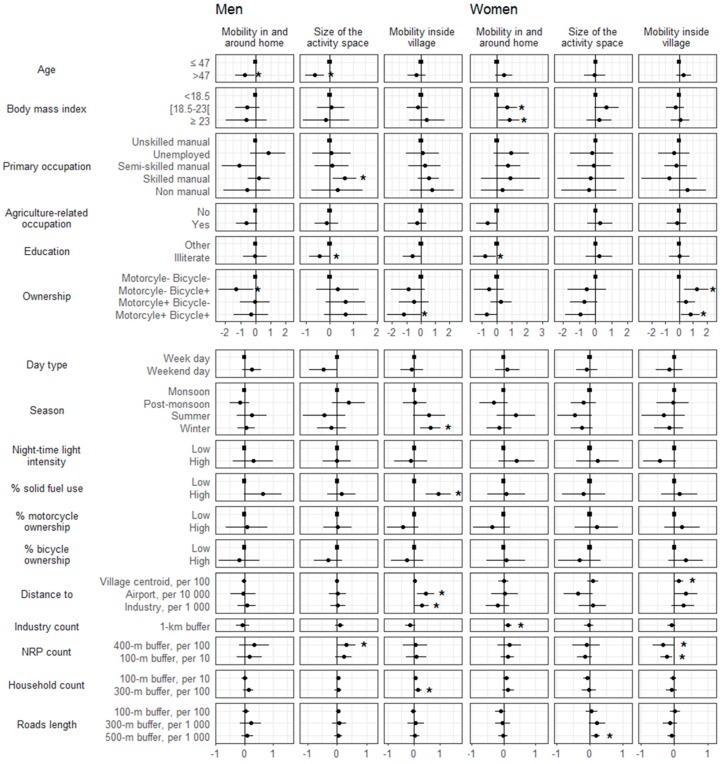
Effects of individual, external, village-level and Geographic Information System (GIS)-derived predictors on the three main dimensions of mobility in men and women. Figures are effect estimates (points) and 95% confidence interval (bars) derived from mixed model with random intercept per participant. Each predictor was investigated individually. Adjustment for age did not change the results. Squares indicate reference categories. Stars indicate statistical significance at the 5% level. Distance, industry count, non-residential place count, household count, and road length are considered as continuous variables. For clarity purposes, only the three dimensions common to men and women are presented. We scaled the dimensions scores so that higher values of the estimates indicated more mobility in the corresponding dimension. Body mass index is expressed in kg/m^2^, distances are expressed in meters. Night-time light intensity was used as marker of village urbanicity. Low and high categories in village-level factors were derived from population median value. Abbreviations: NRP: non-residential place.

Figure 2 shows the effects of individual, external, and contextual characteristics on the three main dimensions of mobility in men and women. Older men showed less mobility in and around home than younger men. The inverse association was observed among women, though not statistically significant. Being illiterate was associated with a smaller size of the activity space in men, and being illiterate was associated with less mobility in and around the home in women. Vehicle ownership (motorcycle or bicycle) led to a bigger size of the activity space in men, but a smaller size of the activity space in women. External factors (season and day type) showed effects on men’s mobility only (Figure 2). Higher village-level urbanicity was associated with more mobility in and around home for both men and women. In men, higher village-level solid fuel use was also associated with more mobility in and around home. Increasing distance to the airport and to industry were associated with more mobility inside village in men and women. The count of non-residential places within residential buffers was, on the contrary, associated with less mobility inside village in women. Road length within residential buffers was associated with a bigger size of the activity space in women, but not men.

## 4. Discussion

Our work addresses an important gap in the literature by describing daily mobility of adults living in a peri-urban area of India using objective GPS data. Our analysis provides three key findings. First, sex was a major determinant of mobility in this population for all indicators considered. Second, we successfully reduced the multidimensionality of mobility into a relatively small number of independent and meaningful dimensions. Third, we identified predictors (age, occupation, education level, vehicle ownership, day type, urbanicity, distance to airport, and distance to non-residential place) related to these mobility dimensions. Overall, our results provide new insights into adults’ mobility in an urbanizing area of India and have direct relevance for assessing exposure to various environmental hazards as well as health-related behaviors in future studies.

Daily mobility or time-activity patterns have been previously investigated, but comparisons with our findings are limited by different methodologies, research questions, study areas and populations. Nonetheless, our results are consistent with the literature regarding predictors of mobility, including: individual (sex, age, and socioeconomic position), contextual (urbanization level and land-use), and external (season and day type) factors.

Sex differences in mobility in the literature showed similar patterns as what we found here, but sex differences are considerably larger in our observations than in prior literature. In European and US cities, men spent only 1 to 1.6 h/day less time indoors at home than women [7,8]. In the Delhi area, a time-budget survey reported men spent 5.6 h indoor at home (in the kitchen, drawing room, or bedroom), which was only 0.6 h less than women [33]. In comparison, we observed a 4-h difference between men (9.4 h) and women (13.4 h) for time spent at home, which likely relates to the household burden carried by women and the associated occupation differences. Age differences in mobility found in the literature mostly related to the differences between childhood, working age, and elderly/retirement and generally showed less mobility with older age [7,34,35,36]. In our population, age showed the opposite effect among men. That result could be explained by the high proportion of older men reporting an agriculture-related occupation. Having an agriculture-related occupation was indeed associated with less mobility in and around home, in particular less time spent at home, in men and women. This could relate to the increase time spent in trips which may reflect the multiple activities performed by agricultural workers. Consistent with other studies, we found that unemployed participants spent more time at the residence [7,8,20]. However, the overall pattern across occupation levels was variable among men, which may be linked to their diverse activities and high variability in mobility. Motorized vehicle use has previously been related to the distance travelled from home and time spent in trips [20,37,38,39]. In our population, household vehicle ownership showed an impact on all mobility dimensions, even among women who did not use such transportation mode (as showed by the maximum speed during trips: 6 km/h). Beyond access to transportation, household vehicle ownership likely is a good proxy for socioeconomic position in our population. Two-wheeled vehicle ownership within the household has been included in the Indian standard living index, among others indicators [40]. Though the relevance of this index has been questioned in the face of the rapid urbanization, the specific vehicle ownership indicator may be still useful as a measure of socioeconomic position.

Urbanization, through the built environment and the availability of infrastructure, influences time spent travelling, distance travelled, and thus the size of the activity space in both high- [20,41,42] and low-/middle-income countries [38,43]. In a suburb of Chennai, India, the mean travel time was 50 min among women, consistent with the 36 min we found, and 1.4 h for men, which is less than the 2.7 h we found [43]. This difference may be explained by the lower level of urbanization in our study area. The travel time in Chennai city center was estimated to be half that of the suburb [43]. Similarly, we found that high village-level urbanicity related to less time spent in trips and more mobility in and around home. The limited spatial resolution of the village-level night-time light intensity may explain the observed lack of significant associations.

Typically for adults in high-income countries, time spent at home changed and activity locations differed from weekends to week days [35,44]. In our population, this was only the case for men (more mobility in and around home and smaller and more circular activity space on weekends). Similarly, in a study in California, day type had stronger effect on men’s time-activity patterns than on women’s [35]. Isaacs et al. found that day type, along with the season, were more important predictors than sex for time-activity patterns [44]. In our population, the post-monsoon related to less time spent at home for women and bigger activity space for men, compared to other seasons. That observation likely relates to the increase in agricultural activities and the greater ease of travel compared to during the monsoon. In contrast, summer season relates to decreased agricultural activities and we observed smaller activity space and more mobility in and around home. According to men’s time-activity diary in our population, reporting indoor at home was lowest during summer season, while outdoor in playground or compound was highest (30% and 23% of daytime, respectively, vs. 45% and 7% in average during other seasons), supporting the idea that season might affect mobility through the balance between time spent indoor and outdoor.

We found a major effect of sex on daily mobility. A difference in mobility between men and women was partially expected because of the cultural context in India. The observed difference, however, was up to four times larger than any of the reported results in the literature. In our data, women stayed close to their residence (on average, they spent 13 h of their daytime within 50 m around home) and had much smaller and more circular activity spaces than men. Women’s mobility was characterized by walking speed trips, short trips, and a small number of activity locations. These findings may be explained by the high household burden carried by Indian women which restricts their mobility [45]. A published analysis of the Indian time-use survey of 1998–1999 reported that women spent an estimated 33.5 h per week on household maintenance and care (vs. 2.9 h for men) [45]. Considering a broader definition that included work inside and outside the home, Lahiri-dutt and Sil estimated household work to be 53.0 h per week for women in India [46]. Women in our study location are predominantly engaged in informal, unpaid, or low-productivity work, which is usually home-based and intermittent, potentially explaining their limited mobility. The Indian time-use survey of 1998–1999 highlighted the predominance of female unpaid/informal workers in agricultural areas, which is consistent with our results [45]. Though, independently of their occupation skill levels, women spent a large amount of time at home on average (>80% of daytime, Figure 1). This large amount of time spent in the home vicinity suggests environmental exposures assessed at the residence may be effective estimates for women in this population, with the caveat that concentrations may differ between indoor and outdoor environments. In contrast, men’s mobility was more complex and variable than women’s. Specifically, men’s mobility variability over time was greater for activity locations indicators, time spent at home, and linear distance from home. The variability over time found in the present study was much larger than in high-income countries population [35,44]. An important characteristic of the workforce in India is the multiple activities performed [45], which may explain the variability over the course of the day and from one day to another in men. The high number of activity locations visited by men and the average of 3 h of trips also support this hypothesis. Another explanation might be the influence of external factors (day type and season) on men’s mobility. Day type and season thus may be important for environmental exposure assessment efforts in this or similar male populations.

Mobility is a multidimensional concept relevant for multiple disciplines and has been measured in different ways depending on the research question of interest. Trip-based definitions consider number of trips, travel time, and mode of transportation and are primarily used for urban planning, built environment, or health research focused on active transport [19,39]. Activity space, the area containing all movements over a determined period of time, can be used to investigate the contextual physical or social effect of the neighborhood on health [47]. Micro-environments, where an individual spends his/her time, are mainly used to understand environmental exposures [48]. As previously proposed [20,21,22], we identified the main dimensions of mobility across multiple existing indicators. In men and women, we identified three dimensions that were also identified in a similar analysis conducted among 2000 participants in Paris, France [20]: the importance of the home vicinity, the size of the activity space, and the shape of the activity space. In the French study, these axes explained 64% of the total variability in mobility; in our population, they explained 61% (men) and 79% (women). Consistent with Perchoux et al., we also observed a dimension related to the volume of activities, although the indicators used were not directly comparable and the proportion of variance explained was lower in our study (<10% vs. 16%) [20]. Indian women’s mobility showed high homogeneity and could be described along three main axes explaining 86% of the total variability. Men’s mobility showed similar features explaining almost 70% of the variability, though it was more complex with the shape of the activity space as a distinct dimension. The median distance travelled from the home dimension in men, needed to reach 80% of variability explained, lacked interpretability but might be related to the size of the activity space dimension. Overall, our results indicate that distinct and meaningful patterns of adult mobility can be identified in a peri-urban population of India. These patterns are likely to be informative in estimating environmental exposures or health behaviors in this or similar populations.

The strengths of our study include objectively measured mobility data using GPS devices, with repeated measurements over different seasons, involving a fairly minimal participant burden. Thanks to the longitudinal design and the homogeneity of the population, we were able to detect relevant associations despite the limited number of participants. The methods used throughout the present analysis (GPS-collected data, automated algorithm to derive activity locations, and data-reduction method) are innovative and generalizable. The principal components analysis identified indicators similar to those identified in an urban European population, suggesting that the relevant indicators to characterize mobility are also fairly generalizable across populations and levels of economic development. The specific findings regarding individual and contextual predictors of these mobility dimensions are likely to be generalizable across peri-urban and rural areas of South India (a population of approximately 250 million).

A number of limitations in the present study should be considered. The homogeneity of the population limited our ability to investigate modification of the relationship between predictors and mobility indicators by specific individual characteristics. We acknowledge that mobility was monitored for one session only for seven participants of the present study population. One day of data collection may not be sufficiently representative of the mobility habits of an individual. However, as our analysis primarily focused on the determinants of mobility in this population and not on individual variability over time, all data available were included. The automated algorithm we used could not distinguish between time spent indoors versus outdoors, a distinction potentially relevant for environmental exposure assessment. In high-income countries, time spent outdoors at home has been estimated to be only 3% in urban areas [7]. Differences are expected for rural and suburban areas [36], as well as for low- and middle-income countries. Using the time-activity diary in our population, we could estimate that 24% to 34% of the time spent at home was spent outdoors. The automated algorithm detected activity locations if time spent was >30 min [15]. By excluding locations visited for short-durations, these were identified as trips. Thus, we likely underestimated the true number of activity locations visited. This aspect likely affected women’s mobility more than men’s, as women are expected to make short trips and stops related to household work and child care. We however believe that aspect did not impact our overall conclusions. Although we cannot exclude the possibility that participants changed their behaviors as a consequence of being monitored, field technicians have noticed that participants adapted quickly to the equipment, thus the influence of these behaviors on our conclusions might be limited. The prediction of transportation mode and identification of sedentary behavior were beyond the scope of the present study objectives. However, the CHAI project collected data from a collocated accelerometer, potentially making possible such analyses in the future.

## 5. Conclusions

In conclusion, we observed strong sex differences in daily mobility in this population, which support the importance of stratified analyses in the investigation of environmental exposures and health behaviors in populations sharing similar cultural and economic contexts. Women spent a large proportion of their daytime close to the residential place, indicating for women the importance of characterizing environment at or very near the home in health studies (e.g., air pollution monitoring in the home), although other approaches (e.g., personal air pollution monitoring) are likely needed for assessing exposures for men. Multiple indicators of mobility could be summarized into independent and meaningful dimensions. The size of the activity space, the importance of the residential place and mobility inside village appear to be useful and generalizable measures of mobility that could be used in future research.

## Figures and Tables

**Table 1:** Indicators of daytime mobility.

Percent daytime spent in:	50-m residential buffer
	100-m residential buffer
	400-m residential buffer
	800-m residential buffer
	1600-m residential buffer
	Village boundaries
Percent daytime spent *:	At home
	In activity locations
	In trips
Activity locations visited:	Total number
	% inside village boundaries
	% inside the 1-standard deviational ellipse
	Average distance from home
Trips:	Number of trips ≥5 min
	Average speed
Activity spaces:	Minimum convex polygon:
	Perimeter
	Surface
	Compactness
	Centroid-to-home distance
	1-standard deviational ellipse:
	Perimeter
	Surface
	Compactness
	Centroid-to-home distance
Linear distance travelled from home:	Mean
	Median

* As identified by the automated algorithm.

**Table 2:** Characteristics of the study population.

N participants	All	Men	Women	*p*-Value
	47	23	24	
Age (years), m (sd)	44 (13.7)	40 (16.1)	49 (8.9)	0.01
min–max	20–65	20–65	27–64	
Number of GPS sessions				
m (sd)	4.1 (1.7)	4.1 (1.8)	4.2 (1.6)	0.62
Only 1 session, n (%)	7 (14.9)	4 (17.4)	3 (12.5)	
GPS recording time (hours), m (sd)	16.3 (0.6)	16.4 (0.7)	16.2 (0.5)	0.04
min–max	13.9–19.2	13.9–19.2	14.2–17.5	
Marital status, married, n (%)	34 (72)	16 (70)	18 (75)	0.01
Education level, illiterate, n (%)	25 (53)	6 (26)	19 (79)	<0.001
Current smoker, n (%)	6 (13)	6 (26)	0	0.03
Primary occupation, n (%)				
Unemployed	4 (9)	2 (9)	2 (8)	0.06
Unskilled manual	26 (55)	9 (39)	17 (71)	
Semi-skilled manual	5 (11)	2 (9)	3 (13)	
Skilled manual	10 (21)	9 (39)	1 (4)	
Non manual	2 (4)	1 (4)	1 (4)	
Agriculture-related occupation, n (%)	26 (55)	8 (35)	18 (75)	0.01
Body mass index (kg/m^2^), n (%)				
<18.5	13 (28)	6 (27)	7 (29)	0.10
18.5−23.0	23 (50)	14 (64)	9 (38)	
≥23.0	10 (22)	2 (9)	8 (33)	
Household ownership, n (%)				
Motorcycle−Bicycle−	7 (15)	3 (13)	4 (17)	0.87
Motorcycle−Bicycle+	7 (15)	4 (17)	3 (12)	
Motorcycle+Bicycle−	23 (49)	12 (52)	11 (46)	
Motorcycle+Bicycle+	10 (21)	4 (17)	6 (25)	

Abbreviation: GPS: Global Positioning System, m: mean, sd: standard deviation. Age calculated on 1 May 2015. Unemployment included housewifes, retired people, and unemployed people. P-values derived from ANOVA (continuous variables) or χ^2^ test (categorical variables) comparing men and women.

**Table 3:** Time spent in different locations and travelled distance from home.

	All	Men	Women
*N Participant-Days*	*192*	*91*	*101*
**Percent daytime spent in:**			
50-m buffer, m (sd)	74 (25.3)	62 (23.4)	84 (22.4)
*min–max*	*22–100*	*22–100*	*38–100*
100-m buffer	76 (25.0)	65 (23.8)	85 (22.1)
	*23–100*	*23–100*	*39–100*
400-m buffer	80 (23.8)	72 (24.0)	87 (21.2)
	*26–100*	*26–100*	*41–100*
800-m buffer	83.5 (22.3)	76 (23.1)	91 (19.0)
	*27–100*	*27–100*	*43–100*
1600-m buffer	88 (20.1)	82 (22.5)	94 (15.9)
	*27–100*	*27–100*	*45–100*
Village boundaries	78 (26.0)	67 (27.0)	87 (21.5)
	*0–100*	*0–100*	*41–100*
**Activity locations visited:**			
Total number, m (sd)	1.6 (1.8)	2.0 (1.7)	1.1 (1.7)
*min–max*	*0–10*	*0–10*	*0–8*
% in village boundaries	26 (39.5)	35 (41.3)	18 (36.2)
	*0–100*	*0–100*	*0–100*
% in 1-standard deviational ellipse	29 (40.1)	42 (42.1)	18 (34.6)
	*0–100*	*0–100*	*0–100*
Average distance from home in km	2.3 (3.7)	3.1 (4.4)	1.0 (1.0)
	*0–19*	*0–19*	*0–3*
**Trips:**			
Number (≥5 min), m (sd)	3.0 (3.2)	4.6 (3.5)	1.5 (1.9)
*min–max*	*0–15*	*0–15*	*0–9*
Average speed in km/h	4.2 (4.9)	6.2 (6.0)	2.2 (1.3)
	*0–30*	*1–30*	*0–6*
**Linear distance travelled from home:**			
Mean distance in km, m (sd)	0.6 (1.2)	1.1 (1.5)	0.2 (0.4)
*min–max*	*0–7*	*0–7*	*0–2*
Median distance in km	0.5 (1.5)	0.9 (2.1)	0.2 (0.6)
	*0–12*	*0–12*	*0–3*

Abbreviation: m: mean, sd: standard deviation. All ANOVA tests comparing men and women were significant at the 5% level.

**Table 4:** Principal component analysis of mobility indicators in men and women.

	Men					Women		
Components Labels	Mobility in and around Home	Size of the Activity Space	Mobility inside Village	Circularity of the Activity Space	Median Distance Travelled from Home	Mobility in and around Home	Size of the Activity Space	Mobility inside Village
*Proportion of total variability explained:*	*27.6%*	*24.3%*	*9.3%*	*9.5%*	*9.8%*	*37.5%*	*41.9%*	*6.4%*
**Percent daytime spent in:**								
50-m buffer	0.90					−0.84	−0.50	
100-m buffer	0.91					−0.83	−0.52	
400-m buffer	0.84				−0.38	−0.78	−0.56	
800-m buffer	0.77				−0.46	−0.56	−0.72	
1600-m buffer	0.52	−0.30			−0.61		−0.89	
Village boundaries	0.77					−0.80	−0.54	
Percent daytime spent *:								
At home	0.78		−0.51			−0.85	−0.49	
In activity locations	−0.42		0.80			0.88	0.40	
In trips	−0.74		−0.38			0.52	0.66	0.31
**Activity locations visited:**								
Total number	−0.66		0.41			0.70	0.44	
% inside 1−sd ellipse	−0.51		0.55			0.65	0.57	
% inside village			0.78					0.64
Average distance from home		0.63		−0.40	0.38	0.52	0.83	
**Trips:**								
Number of trips ≥5 min	−0.82					0.57	0.50	0.45
Average speed		0.60		−0.46		0.37		−0.68
**Minimum convex polygon:**								
Surface		0.94					0.87	
Perimeter		0.94				0.54	0.82	
Compactness				0.89		−0.74		
Centroid–to–home distance		0.65				0.50	0.82	
**1-standard deviational ellipse:**								
Surface		0.96				0.34	0.85	
Perimeter		0.92				0.53	0.83	
Compactness				0.85		−0.86	−0.37	
Centroid–to–home distance	−0.31	0.65			0.39	0.46	0.84	
**Linear distance travelled from home:**								
Mean		0.72			0.55	0.50	0.84	
Median					0.84	0.36	0.78	

* As identified by the automated algorithm. Loading factors were obtained after varimax rotation. Loadings below 0.30 are not presented for clarity. We labeled the components according to the meaning of their high contributing indicators. Abbreviations: 1-sd: 1-standard deviational.

## Supplementary Materials

The following are available online at www.mdpi.com/1660-4601/14/7/783/s1, Table S1: Comparison of the excluded and included participants for the present analysis; Table S2: Village and household characteristics in the study population; Table S3: Activity spaces in the study population; Table S4: Intra-class correlations for time spent in different locations and travelled distance from home; Table S5: Principal component analysis of mobility indicators in the whole population; Table S6: Principal component analysis of mobility indicators in the whole population using only the first session. Figure S1: Effects of individual characteristics on three selected mobility indicators.
