# Supplementary material for: Predictors of Daily Mobility of Adults in Peri-Urban South India
Source: Int J Environ Res Public Health. 2017 Jul 14;14(7):783. doi: 10.3390/ijerph14070783 (PMC5551221; doi:10.3390/ijerph14070783)
Supplement: Supplementary file 1 [file ijerph-14-00783-s001.pdf]

International Journal of
Environmental Research and Public Health

MDPI

Article

Predictors of Daily Mobility of Adults in Peri-Urban South India

Margaux Sanchez [1,2,3,*], Albert Ambros [1,2,3], Maëlle Salmon [1,2,3], Santhi Bhogadi [4], Robin T. Wilson [5], Sanjay Kinra [6], Julian D. Marshall [7] and Cathryn Tonne [1,2,3]

1 Centre for Research in Environmental Epidemiology (CREAL), ISGlobal, Barcelona 08003, Spain; albert.ambros@isglobal.org (A.A.); maelle.salmon@isglobal.org (M.S.); cathryn.tonne@isglobal.org (C.T.)
2 Universitat Pompeu Fabra, Barcelona 08002, Spain
3 CIBER Epidemiología y Salud Pública (CIBERESP), Madrid 28029, Spain
4 Public Health Foundation of India, New Delhi 110070, India; kammilisanthi@gmail.com
5 Geography & Environment, University of Southampton, Highfield Campus, SO17 1BJ, Southampton, UK; robin@rtwilson.com
6 Department of Non-communicable Disease Epidemiology, London School of Hygiene and Tropical Medicine, London WC1E 7HT, UK; sanjay.kinra@lshtm.ac.uk
7 Department of Civil and Environmental Engineering, University of Washington, Seattle, 98195, WA, USA; marshall.julian@gmail.com
* Correspondence: margaux.sanchez@isglobal.org; Tel.: +34-93-227-7365

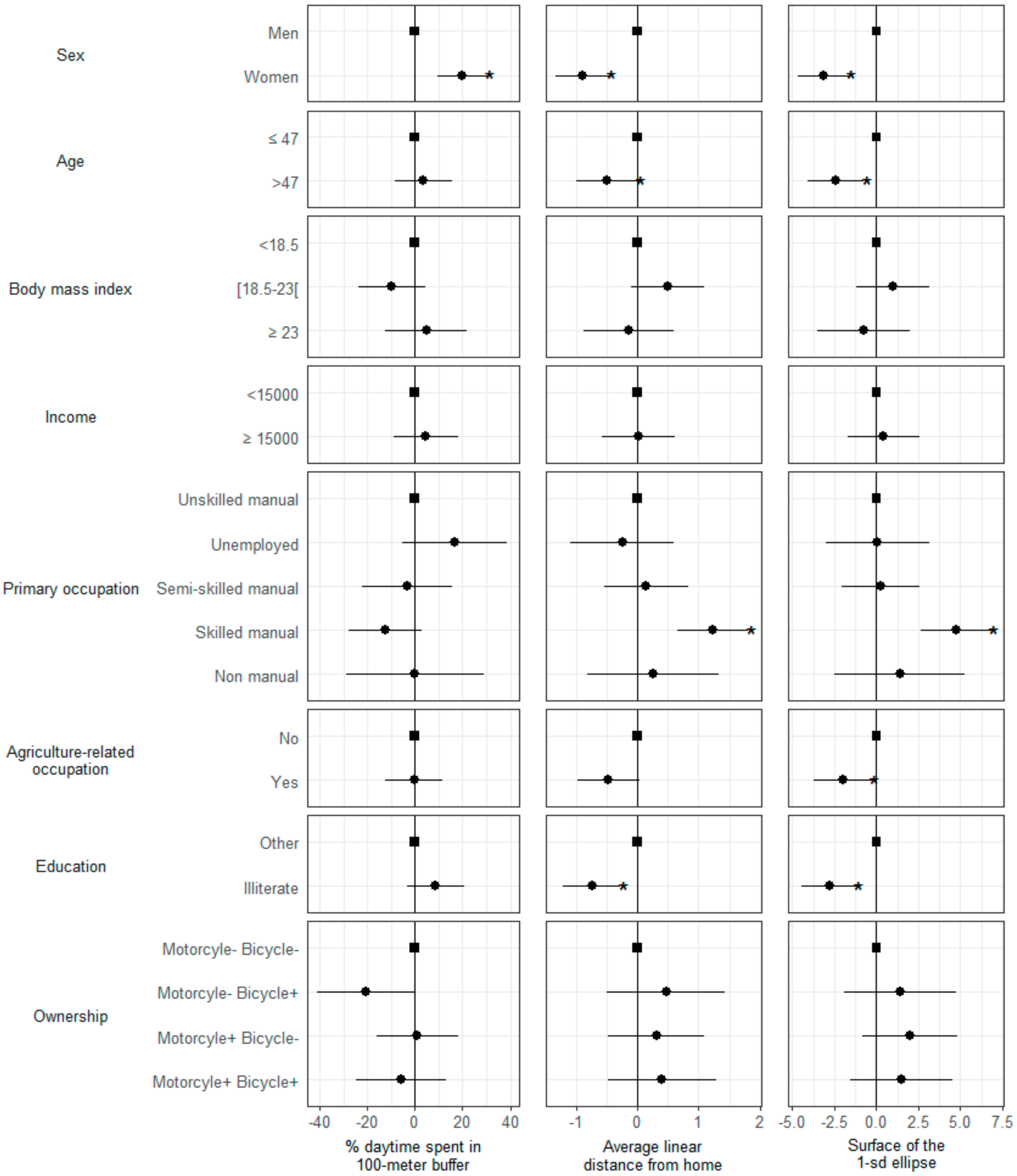

Figure S1. Effects of individual characteristics on three selected mobility indicators.

Figures are effect estimates (points) and 95% confidence interval (bars) derived from a mixed model with random intercept per participant. Each predictor variable was investigated individually. Stars indicate statistical significance at the 5% level. Squares indicate reference categories. Surface of the 1-standard deviational ellipse is expressed in km^2. Linear distance travelled from home is expressed in km. Age is expressed in years, body mass index is expressed in kg/m^2, and income is expressed in Indian Rupee. Abbreviation: sd: standard deviational.

Table S1. Comparison of the excluded and included participants for the present analysis

	**Excluded Participants**	**Included Participants (Study Population)**	***p*-Value**
N	*13*	*47*	
Age (in years), mean (sd)	37 (15.3)	44 (13.7)	0.12
Men, *n* (%)	7 (53.8)	23 (48.9)	1.00
Education level, illiterate, *n* (%)	5 (38.5)	25 (53.2)	0.53
Primary occupation, *n* (%)			
Unemployed	4 (30.8)	4 (8.5)	0.02
Unskilled manual	3 (23.0)	26 (55.3)	
Semi-skilled manual	5 (38.5)	5 (10.6)	
Skilled manual, non-manual	1 (7.7)	12 (25.6)	
Body mass index (kg/m^2), *n* (%)			
<18.5	3 (23.1)	13 (28.3)	0.79
[18.5; 23.0]	6 (46.1)	23 (50.0)	
≥23.0	4 (30.8)	10 (21.7)	
Village-level night-time light intensity, mean (sd)	20.7 (8.8)	17.9 (7.5)	0.24

Abbreviations: sd: standard deviation. Night-time light intensity was used as a marker of village urbanicity. Age was calculated at 1 May 2015. Unemployed included housewife, students, retired, and unemployed people. *p*-values from ANOVA (continuous variables) or χ^2 test (categorical variables) comparing excluded and included participants.

Table S2. Village and household characteristics in the study population.

	All	Men	Women	*p*-Value
N	*47*	*23*	*24*	
**Village characteristics:**				
Number of inhabitants in 2013	3917 (4093)	4245 (4188)	3603 (4065)	0.60
% motorcycle ownership	45.0 (11.0)	45.1 (10.4)	44.9 (11.7)	0.95
% bicycle ownership	54.2 (12.9)	58.2 (10.2)	50.3 (14.1)	0.03
% solid fuel use	54.3 (17.3)	55.0 (17.5)	53.6 (18.5)	0.79
Night-time light intensity in 2012	17.9 (7.5)	18.2 (7.6)	17.5 (7.5)	0.76
Low, *n* (%)	25 (53.2)	12 (52.3)	13 (54.2)	1.00
High, *n* (%)	22 (46.8)	11 (47.8)	11 (45.8)	
**Household characteristics:**				
Distance to primary road (in km)	4.4 (2.8)	4.7 (2.5)	4.1 (3.1)	0.46
	0–10	*1–10*	*0–9*	
Distance to airport (in km)	22.1 (7.7)	23.2 (8.4)	21.1 (7.1)	0.35
	5–35	*6–35*	*5–35*	
Distance to first non-residential place (in meters)	32.1 (27.0)	38.8 (33.2)	25.7 (17.8)	0.10
	1–134	*2–134*	*1–69*	
Distance to village centroid (in meters)	314.5 (372.7)	396.8 (486.3)	235.6 (196.0)	0.14
	19–2437	*24–2437*	*19–678*	
Number of non-residential places in:				
50-meter residential buffer	4.3 (4.9)	2.8 (2.6)	5.7 (6.1)	0.04
	0–22	*0–11*	*0–22*	
100-meter residential buffer	14.0 (12.0)	11.4 (8.0)	16.5 (14.6)	0.15
	0–52	*0–28*	*2–52*	
400- meter residential buffer	98.4 (73.6)	90.0 (65.1)	106.3 (81.5)	0.45
	9–370	*13–334*	*9–370*	
800- meter residential buffer	164.4 (141.0)	165.8 (129.7)	163.1 (153.8)	0.95
	35–696	*51–528*	*35–696*	

Abbreviation: m: mean, sd: standard deviation. Presented figures are mean (standard deviation) and minimum−maximum, unless otherwise stated. Low and high night-time light intensity is derived from population median value. Night-time light intensity was used as an indicator of urbanicity. *p*-values from ANOVA (continuous variables) or χ^2 test (categorical variables) comparing men and women.

Table S3. Activity spaces in the study population.

	All	Men	Women
N participant days	*192*	*91*	*101*
**Minimum convex polygon**			
Surface (in km^2), mean (sd)	3.6 (13.2)	7.5 (18.4)	0.1 (0.3)
min–max	*0–138*	*0–138*	*0–2*
Compactness	0.7 (0.1)	0.7 (0.1)	0.8 (0.1)
	0–1	*0–1*	*0–1*
Perimeter (in km)	5.2 (9.4)	9.5 (12.1)	1.3 (1.8)
	0–71	*0–71*	*0–7*
Centroid to home distance (in km)	0.6 (1.1)	1.1 (1.4)	0.2 (0.4)
	0–10	*0–10*	*0–2*
**1–standard deviational ellipse**			
Surface (in km^2), mean (sd)	1.6 (5.5)	3.2 (7.6)	0.1 (0.1)
min–max	*0–48*	*0–48*	*0–1*
Compactness	0.7 (0.3)	0.8 (0.3)	0.6 (0.2)
	0–1	*0–1*	*0–1*
Perimeter (in km)	4.6 (8.6)	8.3 (11.1)	1.2 (2.2)
	0–54	*0–54*	*0–9*
Centroid to home distance (in km)	0.4 (0.7)	0.7 (0.9)	0.2 (0.4)
	0–5	*0–5*	*0–2*

Abbreviation: sd: standard deviation. All ANOVA tests comparing men and women results were significant at the 5% level.

Table S4. Intra-class correlations for time spent in different locations and travelled distance from home.

	**All**	**Men**	**Women**
N participant days	*192*	*91*	*101*
**Percent daytime spent *:**			
At home	0.51	0.32	0.44
In activity locations	0.35	0.21	0.43
In trips	0.67	0.58	0.34
**Percent daytime spent in:**			
50-meter residential buffer	0.58	0.58	0.41
100-meter residential buffer	0.57	0.60	0.39
400-meter residential buffer	0.53	0.56	0.39
800-meter residential buffer	0.51	0.53	0.34
1600-meter residential buffer	0.54	0.53	0.46
Village boundaries	0.63	0.69	0.42
**Activity locations visited:**			
Total number	0.44	0.40	0.41
% in village boundaries	0.47	0.42	0.49
% in 1-standard deviational ellipse	0.29	0.15	0.33
Average distance from home	0.52	0.50	0.68
**Trips**			
Number (≥5 min)	0.60	0.49	0.42
Average speed	0.52	0.44	0.15
**Linear distance travelled from home:**			
Mean distance	0.37	0.23	0.61
Median distance	0.40	0.35	0.59
**Minimum convex polygon:**			
Surface	0.13	0.05	0.46
Compactness	0.34	0.34	0.31
Perimeter	0.28	0.09	0.56
Centroid-to-home distance	0.14	-0.08	0.63
**1–standard deviational ellipse:**			
Surface	0.12	0.03	0.34
Compactness	0.38	0.19	0.40
Perimeter	0.30	0.14	0.59
Centroid-to-home distance	0.30	0.10	0.65

* As identified by the automated algorithm. As intra-class correlations approaches 0, within-participant variability (namely, variability over time) predominates.

Table S5. Principal component analysis of mobility indicators in the whole population.

Components	1	2	3	4	5
Proportion of total variability explained:	*33.8%*	*25.9%*	*9.8%*	*8.5%*	*5.8%*
**Loading factors**					
**Percent daytime spent in:**					
50-meter buffer	0.92				
100-meter buffer	0.91				
400-meter buffer	0.83		−0.34		
800-meter buffer	0.74		−0.45		
1600-meter buffer	0.53	−0.33	−0.61		
Village boundaries	0.82				
**Percent daytime spent *:**					
At home	0.91				
In activity locations	−0.74				0.47
In trips	−0.71	0.41			−0.35
**Activity locations visited:**					
Total number	−0.79				
% inside 1-sd ellipse		0.68	0.41	−0.34	
% inside village	−0.73				
Average distance from home					0.83
**Trips:**					
Number of trips ≥5 min	−0.81				
Average speed		0.66		−0.42	
**Minimum convex polygon:**					
Surface		0.92			
Perimeter		0.94			
Compactness				0.85	
Centroid-to-home distance	−0.34	0.67			
**1-standard deviational ellipse:**					
Surface		0.95			
Perimeter		0.92			
Compactness	0.51			0.73	
Centroid-to-home distance	−0.37	0.64	0.41		
**Linear distance travelled from home:**					
Mean	−0.32	0.73	0.54		
Median	−0.32		0.84		

* As identified by the automated algorithm. Loading factors obtained after varimax rotation. Loadings below 0.30 are not presented for clarity.

Table S6. Principal component analysis of mobility indicators in the whole population using only the first session.

**Components**	**1**	**2**	**3**	**4**	**5**
Proportion of total variability explained:	*25.7%*	*19.7%*	*17.7%*	*14.2%*	*9.7%*
**Loading factors**					
**Percent daytime spent in:**					
50-meter buffer		0.43	−0.59	0.59	
100-meter buffer		0.52	−0.50	0.59	
400-meter buffer		0.77		0.47	
800-meter buffer		0.80		0.41	
1600-meter buffer		0.74		0.33	
Village boundaries	−0.35	0.60		0.58	
**Percent daytime spent *:**					
At home		0.33	−0.77	0.42	
In activity locations			0.86		
In trips	0.36			−0.78	
**Activity locations visited:**					
Total number			0.74	−0.39	
% inside 1-sd ellipse		−0.40	0.84		
% inside village		0.45	0.79		
Average distance from home	0.72				−0.48
**Trips:**					
Number of trips ≥5 min	0.39		0.39	−0.68	
Average speed	0.73				−0.38
**Minimum convex polygon:**					
Surface	0.92				
Perimeter	0.95				
Compactness					0.80
Centroid-to-home distance	0.70	−0.39			
**1-standard deviational ellipse:**					
Surface	0.93				
Perimeter	0.86			−0.31	
Compactness		0.31	−0.35		0.74
Centroid-to-home distance	0.58	−0.58			−0.34
**Linear distance travelled from home:**					
Mean	0.69	−0.39		−0.40	−0.31
Median		−0.88			

* As identified by the automated algorithm. Loading factors obtained after varimax rotation. Loadings below 0.30 are not presented for clarity. Only the first session data were used, i.e., 47 GPS tracks from 47 unique participants.

© 2017 by the authors. Submitted for possible open access publication under the terms and conditions of the Creative Commons Attribution (CC BY) license (http://creativecommons.org/licenses/by/4.0/).
